# Sex and cardiovascular disease status differences in attitudes and willingness to participate in clinical research studies/clinical trials

**DOI:** 10.1186/s13063-018-2667-7

**Published:** 2018-05-30

**Authors:** Thomas S. Gruca, Wesley J. Hottel, Janine Comstock, Anna Olson, Gary E. Rosenthal

**Affiliations:** 1 University of Iowa, Iowa City, USA; 2 Cognizant Consulting, New York, USA; 3 OMD, New York, USA; 4 Department of Internal Medicine, Wake Forest University, Winston-Salem, NC USA

**Keywords:** Clinical trial, Gender, Cardiovascular disease, Participation, Attitudes

## Abstract

**Background:**

While women are under-represented in research on cardiovascular disease (CVD), little is known about the attitudes of men and women with CVD regarding participation in clinical research studies/clinical trials.

**Methods:**

Patients with CVD (and/or risk factors) and patients with other chronic conditions from Iowa were recruited from a commercial panel. An on-line survey assessed willingness to participate (WTP) and other attitudes towards aspects of clinical research studies.

**Results:**

Based on 504 respondents, there were no differences in WTP in patients with CVD compared to patients with other chronic diseases. Across all respondents, men had 14% lower WTP (relative risk (RR) for men, 0.86, 95% CI, 0.72–1.02). Among patients with CVD, there was no significant difference in WTP between women (RR for women = 1) and men (RR for men, 0.96, 95% CI, 0.82–1.14).

There were no significant differences based on sex or CVD status for attitudes on randomization, blinding, side effects, conflict of interest, experimental treatments or willingness to talk to one’s physician. Women had more favorable attitudes about participants being treated like “guinea pigs” (RR for men, 0.84, 95% CI, 0.73–0.98) and clinical trials being associated with terminally ill patients (RR for men, 0.93, 95% CI, 0.86–1.00).

**Conclusions:**

The findings reported here suggest that the observed lower levels of participation by women are due to factors other than a lower WTP or to women having more negative attitudes towards aspects of study participation. Patients with CVD have similar attitudes and WTP as patients with other chronic conditions.

**Electronic supplementary material:**

The online version of this article (10.1186/s13063-018-2667-7) contains supplementary material, which is available to authorized users.

## Background

Cardiovascular disease (hereafter, CVD) is the leading cause of death for women in the United States [1]. Public relations efforts try to raise the awareness of heart disease in women [2] (e.g., “Go Red for Women”) and more research is being done on heart disease in women [3–5]. Clinical research has identified important sex-related differences with respect to CVD diagnosis, treatment and outcomes [6]. The guidelines for prevention and treatment of CVD are based on the results of clinical research, especially clinical trials [7]. Despite funding mandates [8] and other efforts [9, 10], women continue to be under-represented in clinical research associated with CVD prevention and treatment [11–14].

Most studies documenting the under-representation of women in clinical research on CVD focus on behavior, i.e., enrollment [11–16]. However, research on clinical trials for other conditions (e.g., cancer) suggests that patient attitudes are an important determinant of participation. For example, a national survey [17] found that 45% of respondents felt that cancer patients participating in clinical trials were “treated like ‘guinea pigs’.” On the other hand, a sample of outpatients collected in a Midwest hospital found a high proportion (68%) interested in participating in a clinical trial [18].

Basic psychology suggests that attitudes predict future behavior [19]. Thus, one path to better understanding differences in behavior is to explore variations in attitudes. Consider, for example, the effect of race on participation in cardiology clinical trials. A 2008 study shows that accounting for race-based variations in distrust and perceived harm eliminated the explanatory effect of race on willingness to participate (WTP) in a pharmaceutical clinical trial [20]. Another study compared men’s and women’s WTP in a (hypothetical) CVD prevention clinical trial [21]. In the baseline analysis, women had significantly lower WTP than men. The authors also found that women also had significantly higher levels of distrust in medical professionals compared to men. However, accounting for this difference in attitudes did not eliminate the sex-based variations in WTP.

Given the continuing lack of success in recruiting women for clinical research on CVD, there is a need for more research on women’s attitudes towards participation in such research studies. Researchers studying other patient populations have identified barriers to participation including attitudes towards study randomization [22, 23], potential side effects [24], conflicts of interest [25], etc. The attitudes of CVD patients towards these aspects of clinical research studies are not well-documented in the academic literature. How female and male patients with CVD might differ with respect to these attitudes is also an understudied area.

Recruiting patients for clinical research studies/clinical trials (CRS/CTs) is a challenge not limited to cardiovascular medicine. Researchers studying other chronic diseases also face recruiting difficulties [26–28]. To better understand the attitudes of potential participants in Iowa, the Institute for Clinical and Translational Science (ICTS) at the University of Iowa conducted a general population survey focused on the state of Iowa. This survey was part of a larger effort to promote interest in participating in clinical trials among potential subjects in Iowa. As a first step, the ICTS wanted to better understand attitudes of potential research subjects towards participating in clinical trials and their beliefs about the benefits and harms of participating in clinical trials.

For the analysis presented in this paper, we limited our sample to those patients with at least one of 16 chronic conditions including CVD. These conditions included significant risk factors for CVD such as diabetes, high cholesterol and high blood pressure. Our purpose is to better understand the attitudes of female and male patients with CVD towards various aspects of participation in clinical studies. We included patients with other chronic conditions to better understand what attitudes towards participation are specific to CVD patients and which are shared with other patient groups eligible to participate in clinical research studies/clinical trials (hereafter CRS/CT).

## Methods

### Study sample

We conducted a cross-sectional, on-line survey of adults (aged 18 years and older). Schlesinger Associates, a commercial marketing research firm, recruited respondents from their Inspired Opinions panel. An email invitation directed interested panel members to an on-line survey programmed using Qualtrics. A set of initial questions screened respondents for age and residency in Iowa. Schlesinger Associates compensated respondents upon completion of the survey.

Schlesinger Associates sent a total of 4439 email invitations to prospective participants. Of these, 1250 responded and attempted to complete the survey (28% response rate). This response rate is consistent the reported response rates for recent general population surveys conducted online (average response rate of 24.6%) [29].

A total of 744 respondents completed the survey. Of the respondents completing the survey, 197 (26.5%) reported having none of the 16 chronic conditions of interest (hypertension, diabetes, high cholesterol, heart problems, peripheral vascular disease, stroke, asthma, COPD/emphysema/chronic bronchitis, chronic kidney disease, liver disease, arthritis, osteoporosis, depression, fibromyalgia and cancer). We excluded these patients from the final sample.

**Fig. 1:**
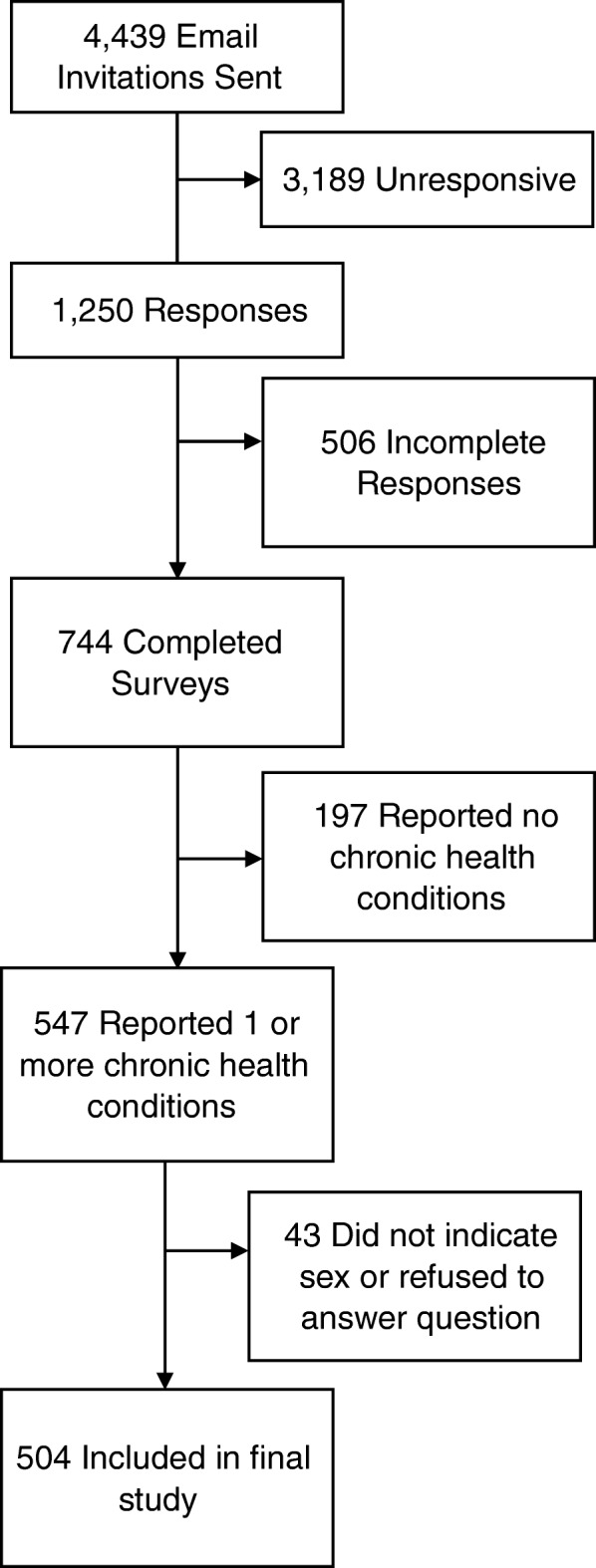
Flowchart of study completion

A total of 43 patients did not disclose their sex. We excluded these respondents from the final sample as well (see Fig. 1). The final sample size was 504 respondents.

The survey was in the field between 15 February and 23 February 2013.

### Instruments

We asked study participants about their knowledge of, past participation in, and various attitudes towards CRS/CTs. We adapted some of the attitude measures from a survey conducted by Mayo Clinic [18]. Based on prior research, we included additional survey items on general attitudes towards clinical research studies/clinical trials. One such item focuses on the impression that patients in clinical trials are “treated like guinea pigs”[17]. Another item inquired whether the participant would be comfortable talking to their physicians about enrolling in a CRS/CT [30]. Additional items asked whether CRS/CTs were for only for terminally ill patients [31] or always involved experimental treatments. For all attitude items, respondents indicated their agreement with this statement on a 5-point Likert scale (Strongly agree, Agree, Neither Agree nor Disagree, Disagree, Strongly disagree).

Willingness to participate was measured using a single item: “I would be interested in participating in clinical research studies/clinical trials related to medical condition(s) I’m interested in (such as conditions I or my friends/family have or may have).” The WTP measure was dichotomized into a positive response (Strongly agree or Agree) and a negative response (Neither Agree nor Disagree, Disagree, or Strongly disagree) [14]. The entire survey is included as an on-line appendix (Additional file 1).

Unlike prior research using written survey forms [14, 18], the location of this item was randomized within the entire set of attitudinal items. This approach reduces the tendency for order effects, exposure effects and other biases associated with written surveys using a single printed form.

To internally validate this measure of WTP with actual behavior, we compared the Likert scale response to the prior experience of the respondent with respect to CRS/CTs. In the survey, respondents were asked if they had been asked to participate in a clinical research study/clinical trial. The possible responses were: “Yes, and I participated,” “Yes, and I declined to participate” and “No.” For those who were asked, we correlated their response (participated or declined to participate) with their WTP response on the 5-point Likert scale using the point-biserial correlation. The non-parametric z-score was 2.54 (*p* <  0.01) which is consistent with past research [14] using WTP measures regarding hypothetical participation in CRS/CTs.

Of the other attitude items, two were reverse coded, i.e., a positive response (Disagree, or Strongly disagree) and a negative response (Neither Agree nor Disagree, Agree or Strongly agree). These are identified in Table 1.

**Table 1:** Responses to survey items on patient attitudes towards and interest in participating in clinical research studies/clinical trials

Label	Question	Positive responses *N*(%)
Random	I find it acceptable to be assigned in a random fashion in a clinical research study/clinical trial.	372(73.8)
Blinded	I find it acceptable to be assigned in a blinded fashion in a clinical research study/clinical trial	388(77.0)
Side effects	Even if I were told that the treatment prescribed to me in a clinical research study/clinical trial has potential side effects, I would still be interested in participating in the clinical research/study	180 (35.7)
Conflict of interest	Clinical research studies/clinical trials sponsored by the pharmaceutical companies are likely to have a conflict of interest	143 (28.4)
Guinea pigs (reversed)	Patients who participate in clinical research studies/clinical trials are treated like guinea pigs	322 (63.9)
Talking to physician	I would feel comfortable talking to my physician about enrolling in a clinical research study/clinical trial	420 (83.3)
Terminally ill (reversed)	Only terminally ill patients participate in clinical research studies/clinical trials	449 (89.1)
Experimental treatments	All clinical research studies/clinical trials involve experimental treatments	124 (24.6)
WTP	I would be interested in participating in clinical trial(s) related to medical condition(s) I’m interested in (e.g., conditions I or my friends/family have or may have)	325 (64.5)

The survey included a question on self-reported health status and items related to respondent demographic characteristics.

This study was approved by the University of Iowa Institutional Review Board (IRB) (#201210760).

### Statistical analysis

Respondents who self-reported having any of the following medical conditions: heart problems (e.g., heart disease, heart failure, etc.), peripheral vascular disease, stroke, high cholesterol, hypertension, or diabetes were classified into a group labeled “CVD.”

Relative risk (RR) analysis was conducted to examine the associations between sex, CVD status and positive WTP. Additional associations between positive WTP and other attitudes related the CRS/CT participation were also estimated. Relative risks were estimated using Poisson regression with robust variance [32].

The baseline sex and CVD status-Stratified Analysis (model 1) were adjusted first for age (18–24, 25–29, 30–39, 40–49, 50–59, 60–64, 65 years and older). An enhanced model (model 2) included age, race (Caucasian (non-Hispanic) or other), household income (US$0–US$24,999, US$25,000–US$49,999, US$50,000–US$74,999, US$75,000–US$100,000, or ≥ US$100,000), marital status (with partner or single), education (less than college graduate or college graduate) and health status (poor/fair, good, very good, excellent).

Further analyses (model 3) examined whether including attitudes towards different aspects of CRS/CTs affected the associations between sex, CVD status and positive WTP. These included attitudes towards randomization, blinding, side effects, conflicts of interest, etc. (The items are listed in Table 1.)

Poisson regression analysis was also used to examine the association of sex or CVD status and the attitude items listed in Table 1.

All analyses were conducted using SAS 9.4. All tests were two-sided.

## Results

Descriptive statistics for the study subjects are provided in Table 2. Our sample is older than the general population. This is likely due to the restriction of this analysis to respondents with a self-reported chronic disease. Chronic diseases, including CVD, tend to be associated with advanced age [33] (the one exception is obesity which is not included in this study).

**Table 2:** Descriptive characteristics of participants

Characteristic	Men (*n* = 195)	Women (*n* = 309)	*P* value^a^	Men proportions	Women proportions
Age (years)			< 0.001		
18–29	4	25		2.1	8.1
30–39	9	46		4.6	14.9
40–49	32	41		16.4	13.3
50–59	56	82		28.7	26.6
60–64	42	51		21.5	16.6
65+	52	63		26.7	20.5
Race			0.15		
Caucasian (non-Hispanic)	186	300		95.4	97.7
Other^c^	9	9		4.6	2.3
Education			0.16		
College graduate	124	176		63.6	57.3
Not a college graduate	71	131		36.4	42.7
Income			< 0.01		
Less than US$25,000	12	51		10.8	16.8
US$25,000 to US$49,999	38	93		19.6	30.6
US$50,000 to US$74,999	58	71		29.9	23.4
US$75,000 to US$99,999	47	47		17.5	15.5
US$100,000+	42	42		22.2	13.8
Marital status			< 0.01		
With partner (married or domestic partnership)	157	214		80.5	69.5
Without partner	38	95		19.5	30.5
Current health status			0.75		
Poor/fair	6	6		3.1	2.0
Good	51	73		26.2	23.8
Very good	114	185		58.5	60.3
Excellent	24	43		12.3	14.0
Cardiovascular disease / risk factors					
Heart problems	31	15	< .0001	15.9	4.9
Peripheral vascular disease	3	1	.30^b^	1.5	0.3
Stroke	5	4	.32^b^	2.6	1.3
Hypertension	76	102	.17	39.0	33.0
High cholesterol	106	129	< .01	54.4	41.8
Diabetes	31	33	.09	15.9	10.7
Any CVD (or risk factor)	151	191	< .001	77.4	61.8

^a^Using chi-square test unless otherwise noted

^b^Fisher’s exact test

^c^African-American (non-Hispanic), Asian, Pacific Islanders, Latino or Hispanic, Native American or Aleut, self-reported “other,” or prefer not to answer

The sample is also more highly educated than the general population. The proportion of adults in Iowa (over 25) holding a bachelor’s degree (or higher) was 26.3% in 2015. In our sample, the percentage was more than double at 60%. This higher level of education is likely due to having an older population and contacting potential respondents through an on-line survey [34]. Similarly, the median income of the survey respondents is higher (US$58,880) than the general population of Iowa (median income of US$54,736). This variation is also likely to be a function of the older age distribution of the sample as well as the use of an on-line panel [35].

There is no significant difference in WTP between men and women in the age-adjusted model (model 1) or in the multivariate model adjusted for other demographic variables (model 2). The results are presented in Table 3.

**Table 3:** Sex, cardiovascular disease (CVD) status and willingness to participate in clinical research studies/clinical trials

	Relative risk (95% CI)			Relative risk (95% CI)		
	Women	Men	*P* value	Non-CVD	CVD	*P* value
Model 1^a^	1 [Reference]	0.93 (0.80–1.07)	.28	1 [Reference]	1.00 (0.86–1.16)	.95
Model 2^b^	1 [Reference]	0.91 (0.79–1.05)	.19	1 [Reference]	0.99 (0.85–1.14)	.86
Model 3^c^	1 [Reference]	0.86 (0.72–1.02)	.09	1 [Reference]	1.04 (0.88–1.24)	.63
Model 3 + random	1 [Reference]	0.85 (0.72–1.01)	.07	1 [Reference]	1.05 (0.88–1.24)	.60
Model 3 + blinded	1 [Reference]	0.85 (0.72–1.00)	.05	1 [Reference]	1.04 (0.88–1.23)	.63
Model 3 + side effects	1 [Reference]	0.85 (0.73–1.00)	.05	1 [Reference]	1.11 (0.95–1.31)	.19
Model 3 + conflict of interest	1 [Reference]	0.85 (0.72–1.01)	.06	1 [Reference]	1.02 (0.86–1.21)	.81
Model 3 + guinea pigs^d^	1 [Reference]	0.92 (0.78–1.09)	.31	1 [Reference]	1.03 (0.87–1.22)	.73
Model 3 + talking to physician	1 [Reference]	0.93 (0.80–1.08)	.36	1 [Reference]	1.05 (0.91–1.23)	.49
Model 3 + terminally ill^d^	1 [Reference]	0.89 (0.75–1.06)	.18	1 [Reference]	1.03 (0.87–1.22)	.73
Model 3 + experimental treatments	1 [Reference]	0.86 (0.72–1.02)	.08	1 [Reference]	1.04 (0.88–1.25)	.63
Stratified characteristics, model 3						
	Non-CVD	CVD	*P* value‖			
Women	1 [Reference]	1 [Reference]	.40			
Men	0.77 (0.57–1.04)	0.96 (0.82–1.14)				

^a^Model 1 is adjusted for age

^b^Model 2 is adjusted for the variables in model 1 plus age, education, annual household income, marital status, race and current health status

^c^Model 3 is adjusted for the variables in model 2 plus CVD status for the primary sex effect estimates and sex for the primary CVD status estimates; model 3 is also adjusted for sex × CVD status interaction

^d^Responses of “Strongly Disagree” and “Disagree” were grouped together

‖*P* values for stratified models are for sex × CVD status interaction

The addition of a sex × CVD status interaction term to the multivariate model (with demographic variables) did not alter the results (model 3). The WTP for men is lower but not significantly different from the WTP for women (model 3: RR, 0.86, 95% CI, 0.72–1.09). The WTP for respondents in the CVD group was not significantly different.

Using model 3, we tested for in WTP across sex and within CVD status. Within the CVD group, the WTP for men is not significantly different from the WTP for women (model 3, Stratified Analysis: CVD group, RR for men, 0.96, 95% CI, 0.82–1.14). The results are similar for the non-CVD group (model 3, Stratified Analysis: non-CVD group, RR for men, 0.77, 95% CI, 0.57–1.04).

The addition of measures of attitudes towards CRS/CTs to model 3 did not affect the difference in WTP for the CVD group compared to those not in the CVD group. None of the differences were significantly different.

The addition of two respondent attitudes to the model of WTP had an impact on the difference in WTP between women and men. The addition of respondent attitudes towards study blinding resulted in a significantly lower WTP for men compared to women (model 3 + blinded: RR for men, 0.85, 95% CI, 0.72–1.00). Similar results were found for the inclusion of attitudes towards side effects (model 3 + side effects: RR for men, 0.85, 95% CI, 0.73–1.00). The results for the other attitudes were not statistically significant at the *p* <  0.05 level.

We compared sex and CVS status differences in the respondents’ attitudes towards CRS/CTs. There were only two significant differences (see Table 4). For men, the proportion with a positive response (Strongly Disagree and Disagree = 1) was significantly lower for the “treated like guinea pigs” (RR for men, 0.84, 95% CI, 0.73–0.98) and the “only for terminally ill patients” RR for men, 0.93, 95% CI, 0.86–1.00) items. Both of these are reverse coded which indicates that men have a significantly less favorable opinion of CRS/CTs with respect to these factors than do the women in the study.

**Table 4:** Sex, cardiovascular disease (CVD) status and positive attitudes associated with participation in clinical trials

	Relative risk (95% CI)			Relative risk (95% CI)		
Attitude^a^	Women	Men	*P* value	Non-CVD	CVD	*P* value
Random	1 [Reference]	0.98 (0.88–1.10)	.79	1 [Reference]	1.03 (0.92–1.16)	.58
Blinded	1 [Reference]	0.99 (0.89–1.20)	.85	1 [Reference]	1.05 (0.94–1.17)	.35
Side effects	1 [Reference]	1.02 (0.79–1.32)	.87	1 [Reference]	0.81 (0.63–1.05)	.11
Conflict of interest	1 [Reference]	1.00 (0.74–1.36)	.99	1 [Reference]	0.92 (0.67–1.25)	.57
Guinea pigs (reversed) ^b^	1 [Reference]	0.84 (0.73–0.98)	.02	1 [Reference]	1.02 (0.88–1.84)	.79
Talking to physician	1 [Reference]	0.95 (0.87–1.04)	.25	1 [Reference]	1.00 (0.91–1.09)	.92
Terminally ill (reversed)^b^	1 [Reference]	0.93 (0.86–1.00)	.05	1 [Reference]	0.98 (0.91–1.05)	.57
Experimental treatments	1 [Reference]	0.85 (0.61–1.19)	.35	1 [Reference]	0.84 (0.59–1.19)	.33

^a^Adjusted for age, marital status, race, education, income and current health status

^b^Disagreement with questionnaire prompt

Comparing attitudes between respondents in the CVD group and those not in the CVD group, no significant differences were found.

## Discussion

Our general population survey of prospective CRS/CT participants in the state of Iowa examined differences in the WTP of men and women, all of whom have a self-reported chronic illness. We found no significant differences between the WTP for men and women. We also did not find significant differences between respondents with CVD and those with one of 10 other chronic illnesses. Most surprising was the finding that the higher WTP for patients in the CVD group was equally high for women as for men. This null finding itself was unexpected given that women are significantly under-represented in clinical trials [36]. Some authors suggest that the low enrollment in studies involving CVD is due to patients having negative attitudes towards various aspects of CRS/CTs such as fear of randomization, mistrust of the researcher, etc. [13]. We found no significant differences between men and women regarding their attitudes towards randomization, blinding, side effects, conflicts of interest or the association of CRS/CTs with experimental treatments. The significant differences in this study were associated with participants being treated like guinea pigs or participation being solely for the terminally ill. However, the results reflected a significantly less positive view of CRS/CTs by the men in our study.

The findings reported here raise some interesting questions about why women are under-represented in CRS/CTs especially in the area of CVD prevention and treatment. The problem does not seem to lie in purely sex-based differences or differences in attitudes towards various potentially negative aspects of clinical research; e.g., randomization, potential conflicts of interest, etc. The question is why – if women are favorably predisposed towards participating in CRS/CTs – do so few actually enroll in these studies? There does seem to be a clear disconnect between the attitudes of women in general (and those with CVD in particular) towards CRS/CTs and their realized participation.

In attempting to explain the continuing failure of researchers to recruit women with CVD for clinical research studies, varying theories are advanced. Some of these are helpful to researchers. For example, prior research suggests that study-based factors account for a great deal of variation in participation [14]. For example, one potential issue is the difference in the age of the onset of CVD in women. Since the onset of CVD is delayed in women [6] (compared to men), studies excluding elderly participants would disproportionately affect women. There is also a role for physicians in educating and counseling women about participation in clinical trials [6].

It may appear cliché to suggest further research on better understanding women’s attitudes and actions regarding participation in CRS/CTs, especially those in the area of CVD prevention and treatment. Simply documenting the problem and speculating about solutions is not working.

Consider, for example, a recent study of women’s participation in 15 cardiology clinical trials at Duke University [14]. In the discussion of patient-level barriers to participation, it was suggested that, “for women especially, there may be a need to arrange child care to attend a study visit.” Given that the median age of the patients in this study was 63 years (I.Q. range of 54–72), it is highly unlikely that arranging child care was a factor for many patients of either gender. Similarly, a recent review article suggested trying to leverage the success of clinical trials for other conditions [6]. For example, the recruiting success of a HIV-treatment study was attributed to, among other factors, the effect of a “feminine logo – a butterfly” to increase recognition of female patients. It is unclear how such an insight may be used to increase women’s participation in clinical research for CVD.

The findings of this study should be viewed in light of its limitations. Our sample was collected from a single Midwestern state with a predominantly non-Hispanic white population (91.3% in 2010 census). While these results might not generalizable to other populations, they are of general interest since a recent national study [37] suggests that non-Hispanic whites have the highest proportion with one or more chronic conditions (63% vs. 58% for non-Hispanic black, 50% for non-Hispanic other and 49% for Hispanic).

Our sample of survey respondents reported themselves as being healthier than the general United States’ population. Overall, 73% identified themselves as having very good or excellent health. This compares to an expected level of 60% using national data (men and women combined, weighted by our sample composition) [38]. Prior research suggests that perceived health may be a barrier to participation in clinical trials [39]. Consequently, the sample of respondents in this study may be more favorably disposed towards CRS/CT participation than the general public.

As noted above, sampling prospective CRS/CT participants through an on-line panel may limit the number of less affluent, less well-educated and less technologically sophisticated respondents. Using a pre-recruited on-line panels for survey research in medicine is an increasing trend [35]. One concern for using this method to study prospective participation in clinical research is the potential for a positive bias towards research in general in the type of person who would participate in an on-line panel. While the extent and nature of such a bias is unknown, we can compare the overall positive response of this study to another survey collected within a single institution with a similar demographic profile of participants [18]. A sample of 400 participants recruited face-to-face at Mayo Clinic (in 2006) found that 68% had a positive WTP. This compares to an overall WTP of 64.5%. While the WTP figures are comparable for these two sampling approaches, it must be noted that face-to-face recruiting of survey participants (which is used in both general population [18] and CVD patient studies [21]) has its own potential problems with introducing bias into survey results.

At the insistence of the IRB, the sponsorship of the study by the University of Iowa was made known to all survey respondents. It is not known whether this information positively or negatively influenced the response rate or the responses by survey participants.

Most importantly, the focal measurement of WTP was based on the response to an attitude scale and not actual enrollment in a clinical research study or clinical trial. No details about the benefits or risks associated with clinical research were presented when the WTP measure was collected. There are many reasons that a person interested in participation does not end up in a clinical trial. Some of these barriers are due to the person (e.g., access to transportation, mobility), some are associated with the requirements of the trial (comorbidity exclusions, etc.). Still others are due to the patient’s relationship with others; e.g., family, spouse, primary care physician. These factors (and more) have been identified in prior research as having an enabling or inhibiting influence on CRS/CT participation.

To improve participation by women in studies on CVD will require different sorts of research than have been done to date. Due to the voluntary nature of enrollment in CRS/CTs, a positive predisposition towards participation is a necessary but not sufficient condition. In other fields, especially cancer care, there has been a concerted effort to study in detail the reasons that patients choose to not enroll in a clinical trial. Some of these qualitative studies [23, 25, 30] served to identify general attitudes that were included in this survey conducted among Iowans with chronic diseases.

Despite the fact that CVD is the leading cause of death for women in the United States, to our knowledge, there been no in-depth qualitative studies of the reasons why women do not enroll in CRS/CTs in the same proportions as men. This study suggests that the typical reasons – concerns with study blinding, randomization, conflicts of interest, etc. – are not different between men and women with chronic diseases (or even within the sub-sample of those with CVD). Therefore, new research must be undertaken to determine if there are unique and addressable barriers in the population of women with CVD that serve as a barrier to participation. Fortunately, there is a recent meta-ethnographic analysis [40] of prior qualitative research (conducted across a range of medical conditions) that identifies several key themes that may be germane to further study of this important and underserved patient population.

## Conclusions

Our study of attitudes towards CRS/CTs among Iowans with chronic conditions shows no significant differences in WTP for women or people with CVD (and/or major risk factors). With respect to attitudes towards various aspects of clinical research, the few observed differences suggest that women have more favorable attitudes. Similarly, patients with CVD have attitudes towards various elements of CRS/CTs similar to those of patients with other chronic conditions. These results contradict the findings from prior research on patient attitudes on WTP in CVD clinical trials. They also are inconsistent with the experience of enrollment by women in studies on CVD prevention and treatment. Better understanding of why women with CVD – who are as favorably disposed to participation as are men – do not enroll in comparable numbers is an important topic for future research to ensure more equitable representation of women in clinical research on CVD.

## Additional file

Additional file 1: Survey ICTS - V2. (DOCX 22 kb)

## Abbreviations

CI: Confidence interval
CRS/CT: Clinical research studies/clinical trials
CVD: Cardiovascular disease
RR: Relative risk
WTP: Willingness to participate
