# Supplementary material for: Sex and cardiovascular disease status differences in attitudes and willingness to participate in clinical research studies/clinical trials
Source: Trials. 2018 May 30;19:300. doi: 10.1186/s13063-018-2667-7 (PMC5975677; doi:10.1186/s13063-018-2667-7)
Supplement: Supplementary file 1 — Survey ICTS - V2. (DOCX 22 kb) [file 13063_2018_2667_MOESM1_ESM.docx]

Survey ICTS - V2

Q1 We invite you to participate in a research study being conducted by investigators from The University of Iowa. The purpose of this study is to assess general opinions and attitudes towards clinical trials / clinical research studies. If you agree to participate, we would like you to answer the survey questions. You are free to skip any questions that you prefer not to answer. It will take approximately 12-15 minutes to complete this survey. We will not collect your name or any identifying information about you.  It will not be possible to link you to your responses on the survey. Taking part in this research study is completely voluntary.  If you do not wish to participate in this study, please close your browser window.  If you have questions about the rights of research subjects, please contact the Human Subjects Office, 105 Hardin Library for the Health Sciences, 600 Newton Rd, The University of Iowa, Iowa City, IA  52242-1098, (319) 335-6564, or e-mail irb@uiowa.edu. Thank you very much for your consideration of this research study.

Q2 Do you agree to read each survey question thoroughly and respond to all questions thoughtfully and honestly?

- Yes (1)
- No (2)

If No Is Selected, Then Skip To End of Block

Q3 Are you at least 18 years old?

- Yes (1)
- No (2)

If No Is Selected, Then Skip To End of Block

Q4 Do you currently reside in Iowa?

- Yes (1)
- No (2)

If No Is Selected, Then Skip To End of Survey

Q5 Please indicate if you have any of the chronic medical conditions listed below. (RANDOMIZE 1-14)

|  | Yes (1) | No (2) |
| --- | --- | --- |
| Hypertension (1) |  |  |
| Diabetes (2) |  |  |
| High Cholesterol (3) |  |  |
| Heart Problems (e.g., heart disease, heart failure, etc.) (4) |  |  |
| Peripheral Vascular Disease (5) |  |  |
| Stroke (6) |  |  |
| Asthma (7) |  |  |
| COPD or Emphysema or Chronic Bronchitis (8) |  |  |
| Chronic Kidney Disease (9) |  |  |
| Arthritis (10) |  |  |
| Osteoporosis (11) |  |  |
| Depression (12) |  |  |
| Fibromyalgia (13) |  |  |
| Cancer (14) |  |  |
| Other chronic medical condition not listed above (15) |  |  |
| I don't have any chronic medical conditions (16) |  |  |

Answer If Please indicate if you have any of the chronic medical co... Other chronic medical condition not listed above - Yes Is Selected

Q6 You indicated that you have a chronic medical condition not included in the list. Please let us know what it is if you feel comfortable sharing this with us.

Q7 Have you ever heard of clinical research studies or clinical trials?

- Yes (1)
- No (2)

Answer If Have you ever heard of clinical research studies or clini... Yes Is Selected

Q8 How did you hear about clinical research studies or clinical trials? Check all that apply.

- From my family physician (1)
- From my specialist physician (e.g., cardiologist, pulmonologist, etc.) (2)
- From a family member (3)
- From a friend (4)
- Other (5)

Answer If How did you hear about clinical research studies or clini... Other Is Selected

Q9 You indicated that you heard about clinical research studies/clinical trials somewhere else. Please let us know how you heard about clinical research studies/clinical trials. Please be specific in your answer.

Q10 Please read the following definition: Clinical research is research that either directly involves a particular person or group of people or uses materials from humans, such as their behavior or samples of their tissue, that can be linked to a particular living person. A clinical trial is one type of clinical research. How familiar are you with clinical research studies/clinical trials?

- Not at All Familiar (1)
- Slightly Familiar (2)
- Somewhat Familiar (3)
- Very Familiar (4)
- Extremely Familiar (5)

Q11 Have you ever been asked to participate in a clinical research study/clinical trial?

- Yes, and I participated (1)
- Yes, and I declined to participate (2)
- No (3)

If No Is Selected, Then Skip To End of Block

Answer If Have you ever been asked to participate in a clinical res... Yes, and I participated Is Selected

Q12 How many clinical research studies/clinical trials have you participated in?

Answer If Have you ever been asked to participate in a clinical res... Yes, and I participated Is Selected

Q13 When was the last time you participated in a clinical research study/clinical trial?

- 0-3 months ago (1)
- 4-6 months ago (2)
- 7-9 months ago (3)
- 10-12 months ago (4)
- More than a year ago (5)

Answer If Have you ever been asked to participate in a clinical res... Yes, and I participated Is Selected

Q14 Please describe the nature of that study/trial in as much detail as possible (for example: was it a survey, a trial that required hospital visits…. etc.).

Answer If Have you ever been asked to participate in a clinical res... Yes, and I participated Is Selected

Q15 Please tell us where this clinical research study/clinical trial took place (i.e., country, state (if applicable), city or metro area).

Country (1)

State (if applicable) (2)

City (or metro area) (3)

Answer If Have you ever been asked to participate in a clinical res... Yes, and I participated Is Selected Or Have you ever been asked to participate in a clinical res... Yes, and I declined to participate Is Selected

Q16 Who asked you to participate in that clinical research study/clinical trial?

- My family physician (1)
- My specialist physician (e.g., cardiologist, pulmonologist, etc.) (2)
- My family member (3)
- My friend (4)
- It was my own decision (5)

Q17 Now we would like to talk to you about your opinion on clinical research studies/clinical trials in general. You will not be asked to participate in a clinical research studies/clinical trials;  however, we would appreciate your honest feedback and opinions about them. (RANDOMIZE)

Q18 I would be interested in participating in clinical trial(s) related to medical condition(s) I'm interested in (e.g., conditions I or my friends/family have or may have).

- Strongly Disagree (1)
- Disagree (2)
- Neither Agree nor Disagree (3)
- Agree (4)
- Strongly Agree (5)

Q19 I find it appropriate for a medical researcher to contact me by phone to inform me about a research project.

- Strongly Disagree (1)
- Disagree (2)
- Neither Agree nor Disagree (3)
- Agree (4)
- Strongly Agree (5)

Q20 I find it appropriate for a medical researcher to contact me by mail to inform me about a research project.

- Strongly Disagree (1)
- Disagree (2)
- Neither Agree nor Disagree (3)
- Agree (4)
- Strongly Agree (5)

Q21 Most of the current treatments in medicine are based on evidence from clinical trials/clinical research studies.

- Strongly Disagree (1)
- Disagree (2)
- Neither Agree nor Disagree (3)
- Agree (4)
- Strongly Agree (5)

Q22  In clinical research studies/clinical trials, participants are often divided into groups in a random fashion (like by the flip of a coin). Both the participant and the medical researcher have no control over whether the participant gets the study treatment or placebo (sugar pill). I find it acceptable to be assigned in a random fashion in a clinical research study/clinical trial.

- Strongly Disagree (1)
- Disagree (2)
- Neither Agree nor Disagree (3)
- Agree (4)
- Strongly Agree (5)

Q23 It would be important for me to be informed by the medical researcher about the results of the clinical research study/clinical trial in which I participated.

- Strongly Disagree (1)
- Disagree (2)
- Neither Agree nor Disagree (3)
- Agree (4)
- Strongly Agree (5)

Q24 In clinical research studies/clinical trials, participants are often divided into groups in a blinded fashion. Both the participant and the medical researcher do not know whether the participant gets the study treatment or placebo (sugar pill). I find it acceptable to be assigned in a blinded fashion in a clinical research study/clinical trial.

- Strongly Disagree (1)
- Disagree (2)
- Neither Agree nor Disagree (3)
- Agree (4)
- Strongly Agree (5)

Q25 Even if I were told that the treatment prescribed to me in a clinical research study/clinical trial has potential side effects, I would still be interested in participating in the clinical research/study.

- Strongly Disagree (1)
- Disagree (2)
- Neither Agree nor Disagree (3)
- Agree (4)
- Strongly Agree (5)

Q26 I am satisfied with the information I currently have about clinical research studies/clinical trials involving medical condition(s) I am interested in (e.g., conditions I or my friends/family have or may have).

- Strongly Disagree (1)
- Disagree (2)
- Neither Agree nor Disagree (3)
- Agree (4)
- Strongly Agree (5)

Q27 I expect my treating physician to inform me about current clinical research studies/clinical trials in the medical condition(s) of my interest (such as conditions that I or my family/friends have or may have).

- Strongly Disagree (1)
- Disagree (2)
- Neither Agree nor Disagree (3)
- Agree (4)
- Strongly Agree (5)

Q28 All reasonable precautions for my safety are likely to be taken in a University of Iowa Hospitals and Clinics (UIHC) –sponsored clinical research study/clinical trial.

- Strongly Disagree (1)
- Disagree (2)
- Neither Agree nor Disagree (3)
- Agree (4)
- Strongly Agree (5)

Q29 All reasonable precautions for my privacy are likely to be taken in a University of Iowa Hospitals and Clinics (UIHC) –sponsored clinical research study/clinical trial.

- Strongly Disagree (1)
- Disagree (2)
- Neither Agree nor Disagree (3)
- Agree (4)
- Strongly Agree (5)

Q30 Clinical research studies/clinical trials sponsored by the pharmaceutical companies are likely to have a conflict of interest.

- Strongly Disagree (1)
- Disagree (2)
- Neither Agree nor Disagree (3)
- Agree (4)
- Strongly Agree (5)

Q31 Patients who participate in clinical research studies/clinical trials are treated like guinea pigs.

- Strongly Disagree (1)
- Disagree (2)
- Neither Agree nor Disagree (3)
- Agree (4)
- Strongly Agree (5)

Q32 I would feel comfortable talking to my doctor about enrolling in a clinical research study/clinical trial.

- Strongly Disagree (1)
- Disagree (2)
- Neither Agree nor Disagree (3)
- Agree (4)
- Strongly Agree (5)

Q33 Only terminally ill patients participate in clinical research studies/clinical trials.

- Strongly Disagree (1)
- Disagree (2)
- Neither Agree nor Disagree (3)
- Agree (4)
- Strongly Agree (5)

Q34 Chronically ill patients often participate in clinical research studies/clinical trials.

- Strongly Disagree (1)
- Disagree (2)
- Neither Agree nor Disagree (3)
- Agree (4)
- Strongly Agree (5)

Q35 Healthy people, i.e., people without any current medical conditions, can participate in clinical research studies/clinical trials.

- Strongly Disagree (1)
- Disagree (2)
- Neither Agree nor Disagree (3)
- Agree (4)
- Strongly Agree (5)

Q36 All clinical research studies/clinical trials involve invasive medical procedures.

- Strongly Disagree (1)
- Disagree (2)
- Neither Agree nor Disagree (3)
- Agree (4)
- Strongly Agree (5)

Q37 All clinical research studies/clinical trials involve experimental treatments.

- Strongly Disagree (1)
- Disagree (2)
- Neither Agree nor Disagree (3)
- Agree (4)
- Strongly Agree (5)

Answer If Have you ever been asked to participate in a clinical res... Yes, and I declined to participate Is Selected

Q38  You indicated that you declined to participate in a clinical research study/clinical trial. How influential were the following considerations in your decision NOT to participate?

|  | Not at all Influential (1) | Slightly Influential (2) | Somewhat Influential (3) | Moderately Influential (4) | Very Influential (5) | Extremely Influential (6) |
| --- | --- | --- | --- | --- | --- | --- |
| I didn't feel comfortable sharing my personal information (1) |  |  |  |  |  |  |
| I didn’t feel comfortable contributing biological material (e.g., blood or tissue sample) (2) |  |  |  |  |  |  |
| Undergoing tests and procedures that might be invasive (3) |  |  |  |  |  |  |
| Time constraints (4) |  |  |  |  |  |  |
| Risk of mistreatment by the research team, such as exploitation, assault on my human rights and dignity (5) |  |  |  |  |  |  |
| Interests of researchers might not be the same as mine (6) |  |  |  |  |  |  |
| I didn’t understand how the clinical research study is designed or conducted (7) |  |  |  |  |  |  |
| Lack of compensation (8) |  |  |  |  |  |  |
| I felt pressured by my family physician / specialist physician to participate in a clinical research study/clinical trial (9) |  |  |  |  |  |  |
| I was afraid of possible side effects (10) |  |  |  |  |  |  |
| I didn't see any benefit for me (11) |  |  |  |  |  |  |
| Travel to study site (12) |  |  |  |  |  |  |

Q39 Now we would like to talk to you about your opinion on medical issues in general.

Q40 What is your primary source of information on medical conditions?

- Internet (1)
- My physician (2)
- Friends/Family (3)
- My specialist physician (e.g., cardiologist, pulmonologist, etc.) (4)
- Other (5)

Answer If What is your primary source of information on medical con... Internet Is Selected

Q41 You indicated that you use online resources to obtain information on medical conditions. Please tell us what websites, forums, online magazines/newspapers, or search engines you use.

Answer If What is your primary source of information on medical con... Other Is Selected

Q42 Please tell us how you find information on medical conditions. (e.g., specific magazines, newspapers, bulletin boards, etc.).

Q43 If you are interested in a clinical research study/clinical trial, where would you look for more information?

- Internet (1)
- My physician (2)
- Friends/Family (3)
- My specialist physician (e.g., cardiologist, pulmonologist, etc.) (4)
- Other (5)

Answer If If you are interested in a clinical research study, where... Internet Is Selected

Q44 You indicated that you would use online resources to obtain information on clinical research studies/clinical trials. Please tell us how/where you would do that (e.g., specific websites, forums, online magazines/newspapers, search engines).

Answer If If you are interested in a clinical research study, where... Other Is Selected

Q45 Please indicate where/how you would find information about clinical research studies/clinical trials (e.g., specific magazines, newspapers, bulletin boards, etc.).

Q46 Details about clinical trials can be easily accessed without charge at clinicaltrials.gov (all U.S. trials). Were you aware of this information?

- Yes (1)
- No (2)

Q47 Imagine there were a clinical trial testing a treatment for a medical condition you are interested in.  How influential are the following considerations in your decision whether or not to participate in this trial? (RANDOMIZE)

|  | Not at all Influential (1) | Slightly Influential (2) | Somewhat Influential (3) | Moderately Influential (4) | Very Influential (5) | Extremely Influential (6) |
| --- | --- | --- | --- | --- | --- | --- |
| Travel/distance from home (1) |  |  |  |  |  |  |
| Number of visits required (2) |  |  |  |  |  |  |
| Time requirements (3) |  |  |  |  |  |  |
| Access to state-of-the-art care and treatments (4) |  |  |  |  |  |  |
| Access to first-rate specialists (5) |  |  |  |  |  |  |
| Reputation of the organization sponsoring/conducting clinical research study/trial (6) |  |  |  |  |  |  |
| My doctor’s recommendation/advice to participate (7) |  |  |  |  |  |  |
| Financial compensation (8) |  |  |  |  |  |  |
| Helping others with the same condition; doing good for mankind (9) |  |  |  |  |  |  |

Q48  How far would you travel to participate in a clinical research study or clinical trial that deals with a medical condition that you are interested in?

- less than 50 miles (1)
- 50 - 100 miles (2)
- 101 - 150 miles (3)
- 151 - 200 miles (4)
- 201 - 250 miles (5)
- more than 250 miles (6)

Q49 Please read the following statement: At the University of Iowa Hospitals and Clinics (UIHC), faculty and staff conduct clinical research studies to look for new and better ways to prevent, detect, diagnose or treat a disease. Would you consider going to UIHC for a clinical research study/trial on a medical condition you are interested in (such as I or my friends/family have or may have)?

- Strongly Disagree (1)
- Disagree (2)
- Neither Agree nor Disagree (3)
- Agree (4)
- Strongly Agree (5)

Answer If Please read the following statement: At the University o... Strongly Agree Is Selected Or Please read the following statement: At the University o... Agree Is Selected Or Please read the following statement: At the University o... Neither Agree nor Disagree Is Selected

Q50  Which of the following would you prefer as compensation for your time and involvement in a University of Iowa Hospitals and Clinics (UIHC)-sponsored clinical research study/clinical trial? Please check all that apply.

- Modest monetary compensation (1)
- Donation to charity of my choice (2)
- Travel and accommodation reimbursement (3)
- Parking reimbursement (4)
- University of Iowa souvenir (5)
- Other (6)

Answer If &nbsp;Which of the following would you prefer as compensa... Other Is Selected

Q51 You indicated that you would prefer a different type of compensation for your time and involvement in a University of Iowa Hospitals and Clinics (UIHC)-sponsored clinical research study/clinical trial. Please specify the type of compensation.

Q52 I would consider allowing my child to participate in a clinical research study/clinical trial.

- Strongly Disagree (1)
- Disagree (2)
- Neither Agree Nor Disagree (3)
- Agree (4)
- Strongly Agree (5)
- Not applicable. I do not have children under 18. (6)

If Not applicable. I do not ha... Is Selected, Then Skip To End of Block

Answer If I would consider allowing my child to participate in a cl... Strongly Disagree Is Selected Or I would consider allowing my child to participate in a cl... Disagree Is Selected

Q53 You indicated that you would not consider allowing your child to participate in a clinical research study/clinical trial. Please tell us why.

Answer If I would consider allowing my child to participate in a cl... Neither Agree Nor Disagree Is Selected

Q54 You indicated that you don't have a strong opinion on whether you would consider allowing your child to participate in a clinical research study/clinical trial. Please tell us why.

Answer If I would consider allowing my child to participate in a cl... Agree Is Selected Or I would consider allowing my child to participate in a cl... Strongly Agree Is Selected

Q55 You indicated that you would consider allowing your child to participate in a clinical research study/clinical trial. Please tell us why.

Q56 These last few questions are for clarification purposes only.

- Male (1)
- Female (2)
- Prefer not to answer (3)

Q58 What is your age?

- 18-24 (1)
- 25-29 (2)
- 30-39 (3)
- 40-49 (4)
- 50-59 (5)
- 60-64 (6)
- 65+ (7)

Q59 Which best describes your marital status?

- Single, never married (1)
- Married (2)
- Separated/divorced/widowed (3)
- Domestic partnership (4)
- Prefer not to answer (5)

Q60 How far from Iowa City do you live?

- less than 50 miles (1)
- 50 - 100 miles (2)
- 101-150 miles (3)
- 151 - 200 miles (4)
- 201 - 250 miles (5)
- more than 250 miles (6)

Q61 How would you rate your overall health?

- Very Poor (1)
- Poor (2)
- Fair (3)
- Good (4)
- Very Good (5)

Q62 Which of the following racial groups best describes you?

- African-American (non-Hispanic) (1)
- Asian (2)
- Pacific Islanders (3)
- Caucasian (non-Hispanic) (4)
- Latino or Hispanic (5)
- Native American or Aleut (6)
- Other (7)
- Prefer not to answer (8)

Q63 What is the highest level of formal education you have completed?

- Completed some high school or less (1)
- High school graduate (2)
- Completed some college (3)
- College degree (4)
- Completed some postgraduate (5)
- Master's degree (6)
- Doctorate, law, or professional degree (7)

Q64 What best describes your current level of income?

- Less than $25,000 (1)
- $25,000 to $50,000 (2)
- $50,000 to $75,000 (3)
- $75,000 to $100,000 (4)
- $100,000+ (5)

Q65 Is there anything else you would like to tell us? Thank you very much for your time.
